# (*E*)-1,3-Bis(anthracen-9-yl)prop-2-en-1-one: crystal structure and DFT study

**DOI:** 10.1107/S2056989018003791

**Published:** 2018-03-09

**Authors:** Dian Alwani Zainuri, Ibrahim Abdul Razak, Suhana Arshad

**Affiliations:** a X-ray Crystallography Unit, School of Physics, Universiti Sains Malaysia, 11800 USM, Penang, Malaysia

**Keywords:** crystal structure, chalcone, absorption spectra, HOMO–LUMO, Hirshfeld surface

## Abstract

The title compound, C_31_H_20_O, was synthesized using a Claisen–Schmidt condensation. The enone group adopts an *s*-*trans* conformation and the anthracene ring systems are twisted at angles of 85.21 (19) and 83.98 (19)° from the enone plane. In the crystal, mol­ecules are connected into chains along [100] *via* weak C—H⋯π inter­actions. The observed band gap of 3.03 eV is in excellent agreement with that (3.07 eV) calculated using density functional theory (DFT) at the B3LYP/6–311++G(d,p) level. The Hirshfeld surface analysis indicates a high percentage of C⋯H/H⋯C (41.2%) contacts in the crystal.

## Abstract 2

In the crystal, mol­ecules are connected into chains along [100] *via* weak C—H⋯π inter­actions. The observed band gap of 3.03 eV is in excellent agreement with that (3.07 eV) calculated using density functional theory (DFT) at the B3LYP/6–311++G(d,p) level. The Hirshfeld surface analysis indicates a high percentage of C⋯H/H⋯C (41.2%) contacts in the crystal.

## Chemical context   

Anthrancene and its derivatives constitute a very well-known class with inter­esting photophysical properties and they are used extensively in the design of luminescent chemosensors and switches (Montalti *et al.*, 2000 ▸). A chalcone mol­ecule with a π-conjugated system provides a large charge-transfer axis with appropriate substituent groups on the terminal aromatic rings. Strong inter­molecular charge transfer (ICT) will give rise to second harmonic generation (SHG) efficiency and this may enhance the non-linear optical (NLO) properties (D’silva *et al.*, 2011 ▸). Furthermore, π-conjugated mol­ecular materials with fused rings are the focus of considerable inter­est in the emerging area of organic electronics, since the combination of good charge-carrier mobility and high stability may lead to potential optoelectronic applications (Wu *et al.*, 2010 ▸). As part of our work in this area, we now report the synthesis and combined experimental and theoretical studies of the title compound, (I).
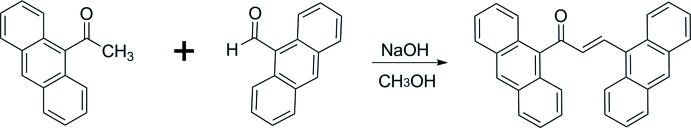


## Structural commentary   

**Figure 1:**
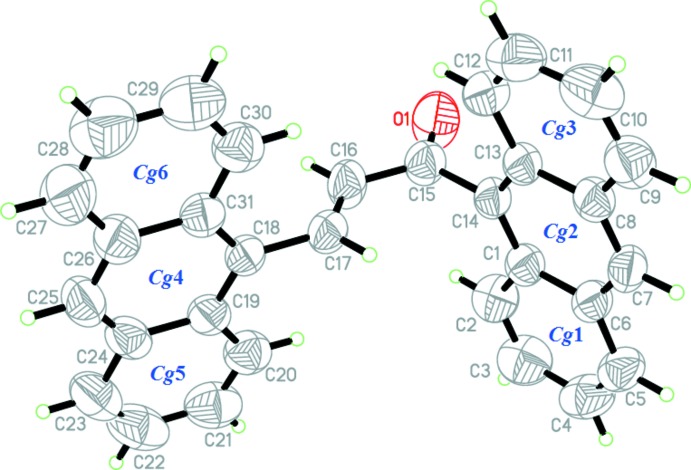
The mol­ecular structure of (I) showing 50% displacement ellipsoids.

The mol­ecular structure of (I) is shown in Fig. 1 ▸ (for the optimized structure, see Fig. S1 in the Supporting information). The structure consists of two anthracene rings (Anth *A* and Anth *B*) . Anth *A* is formed by the aromatic rings labeled as *Cg*1(C1–C6), *Cg*2(C1/C6–C8/C13/C14) and *Cg*3(C8–C13). Anth *B* consists of *Cg*4(C18/C19/C24–C26/C31, *Cg*5(C19–C24) and *Cg*6(C26–C31).

The C—C distances in the central ring of the anthracene units show little variation compared to the other rings (Anth *A*: C20—C21, C22—C23, C27—C28 and C29—C30; Anth *B*: C2—C3, C4—C5, C9—C10 and C11—C12), which are much shorter. These observations are consistent with an electronic structure for the anthracene units where a central ring displaying aromatic delocalization is flanked by two isolated diene units (Glidewell & Lloyd, 1984 ▸). Both theoretical and experimental structures exist in an *E* configuration with respect to the C16=C17 double bond [experimental = 1.291 (2) Å and DFT (see below) = 1.34 Å].

The enone moiety (O1/C15–C17) shows an *s*-*trans* configuration with the O1—C15—C16—C17 torsion angle being −179.19 (19) and 179.64° in the experimental and calculated structures, respectively. Additionally, the enone moiety [O1/C15–C17, maximum deviation of 0.0039 (18) Å at C16] forms dihedral angles of 85.21 (19) and 83.98 (19)° with the Anth *A* [C1–C14, maximum deviation of 0.103 (2) Å at C11] and Anth *B* [C18–C31, maximum deviation of 0.016 (3) Å at C27] groups, respectively. The large dihedral-angle deviation indicates that the possibility for electronic effects between the anthracene units through the enone moiety has decreased (Jung *et al.*, 2008 ▸). This is in contrast with the mol­ecular structure of (*E*)-1-(anthracen-9-yl)-3-(2-chloro-6-fluoro­phen­yl)prop-2-en-1-one (Abdullah *et al.* 2016 ▸), which shows the enone moiety locked in an *s*-*cis* configuration because of the intra­molecular hydrogen bond. Furthermore, the bulkiness of the anthracene ring gives rise to a highly twisted structure at both terminal rings. Compound (I) is twisted at the C17—C18 and C14—C15 bonds with C16—C17—C18—C19 and C1—C14—C15—C16 torsion angles of 84.0 (2) and 93.65 (19)°, respectively (see Fig. S2 in the Supporting information). The corresponding torsion angles for the DFT study are 48.01 and 94.05°, respectively. We propose that the torsion-angle difference of about 35.9° between the experimental and DFT studies are the result of the formation of inter­molecular C—H⋯π inter­actions involving the anthracene units. The observed inter­molecular inter­actions in the crystal packing are the main cause of the angle difference when this inter­action is not taken into consideration during the optimization process.

## Supra­molecular features   

**Figure 2:**
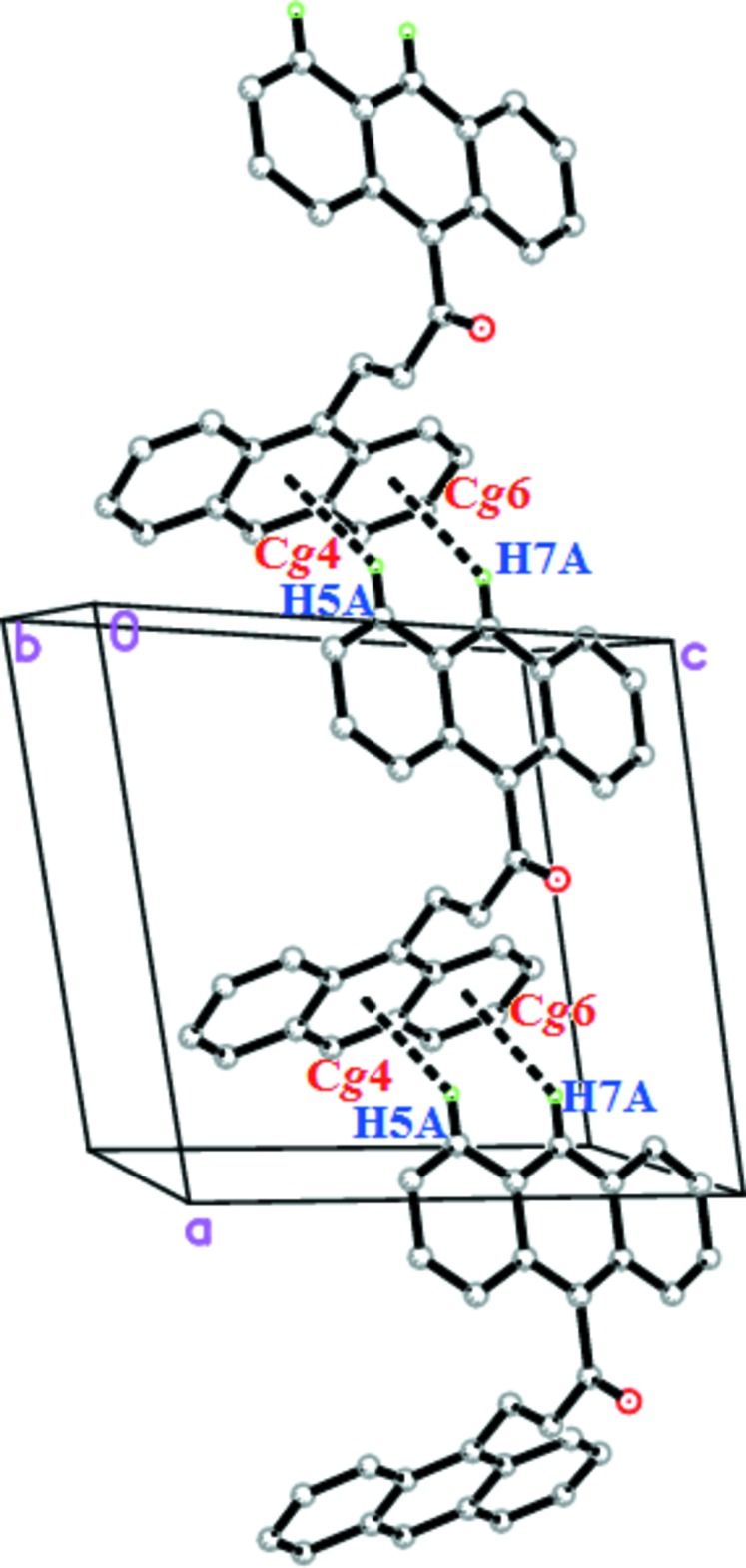
The weak C—H⋯π inter­actions in the crystal of (I).

In the crystal of (I), C—H⋯π inter­actions are mainly responsible for the packing. Two C—H⋯π inter­actions (Fig. 2 ▸ and Table 1 ▸) occur between anthracene rings (Anth *A* and Anth *B*), connecting the mol­ecules into infinite zigzag chains propagating along the [100] direction.

## Theoretical chemistry study   

The optimization of the mol­ecular geometries leading to energy minima was achieved using DFT [with Becke’s non-local three parameter exchange and the Lee–Yang–Parr correlation functional (B3LYP)] with the 6-311++G (d,p) basis set as implemented in *Gaussian09* program package (Frisch *et al.*, 2009 ▸). The selected bond lengths and angles of the optimized structure in comparison to the experimental values are presented in Table S2 in the Supporting information and the optimized structure is presented in Figure S1. Agreement between experimental and calculated geometrical data is generally good and any deviations may be ascribed to the fact that the optimization is performed in an isolated condition, whereas the crystal environment affects the mol­ecular geometry (Ramya *et al.*, 2015 ▸).

## Absorption spectrum and frontier mol­ecular orbitals   

**Figure 3:**
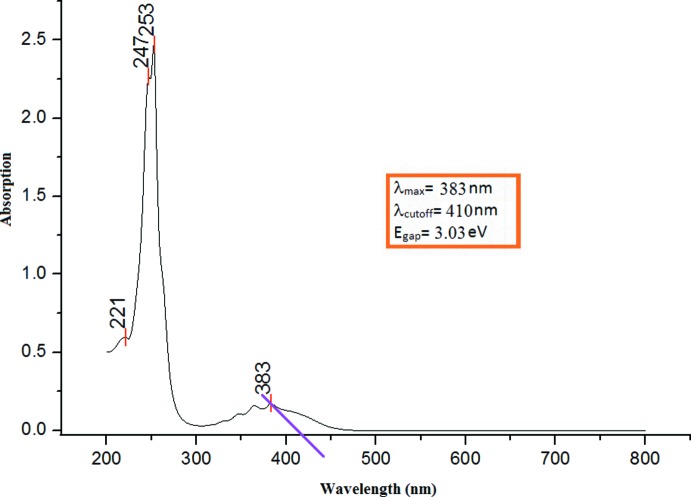
UV–Vis absorption spectra of (I).

The longest wavelength absorption maxima for (I) is observed in the UV region at 383 nm as shown in Fig. 3 ▸. The TD–DFT calculation at the B3LYP/6-311G++(d,p) level shows that this feature is due to an electronic transition from the highest occupied mol­ecular orbital (HOMO) to the lowest unoccupied mol­ecular orbital (LUMO). In the ground state (HOMO), the charge densities are mainly delocalized over the anthracene rings and the enone moiety, while in the LUMO state, the charge densities are accumulated on the Anth *A* and enone moiety (see Fig. S3 in the Supporting information). The calculated λ_max_ of 390 nm is shifted from the experimental value, which may be attributed to solvent effects, compared to the gas-phase calculation.

The HOMO–LUMO energy gap (Fig. S3) relates to the chemical activity of the mol­ecule (Kosar & Albayrak, 2011 ▸). The predicted energy gap of 3.07 eV shows excellent agreement with the estimated experimental energy gap of 3.03 eV. These optical band-gap values indicate the potential suitability of this compound for optoelectronic applications, as previously reported by Prabhu *et al.* (2016 ▸). Additionally, Nietfeld *et al.* (2011 ▸) compared the structural, electrochemical and optical properties of fused-ring and non-fused ring compounds, indicating that fused rings have lower band gaps than other structures.

## Hirshfeld Surface analysis   

**Figure 4:**
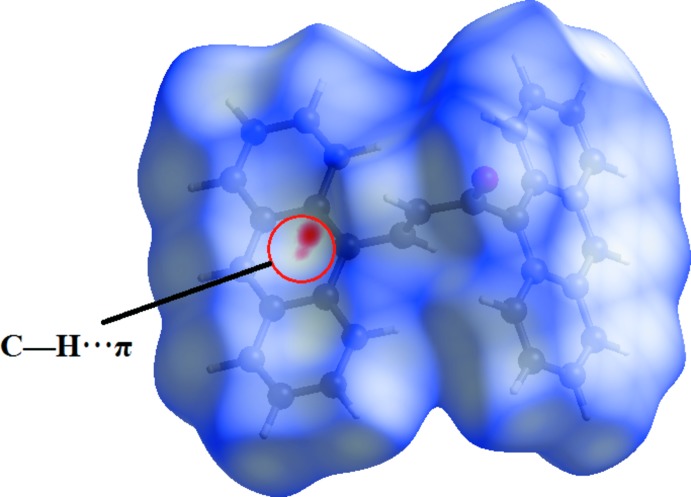
View of the Hirshfeld surfaces mapped over *d_norm_* for (I).

**Figure 5:**
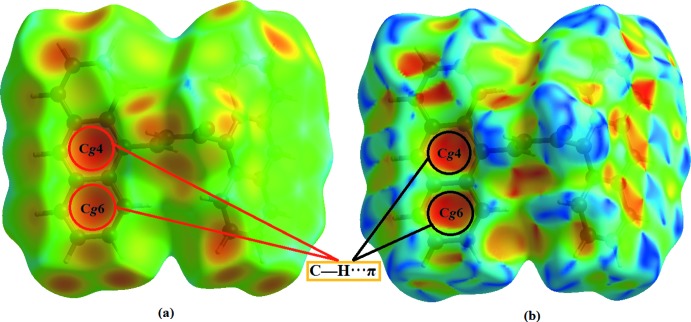
View of the Hirshfeld surfaces for (I) mapped over (*a*) *d_e_* and (*b*) shape-index with the pale-orange spot within the red circles showing the presence of the C—H⋯π inter­actions.

Fig. 4 ▸ shows the Hirshfeld surface mapped over *d_norm_*. As expected, the *d_norm_* surfaces reveal the C—H⋯π inter­molecular inter­action as a large depression (bright-red spot). The presence of this C—H⋯π inter­action is also indicated through the combination of pale-orange and bright-red spots that are present on the Hirshfeld surfaces mapped over *d_e_* (Fig. 5 ▸ *a*) and shape-index (Fig. 5 ▸ *b*).

**Figure 6:**
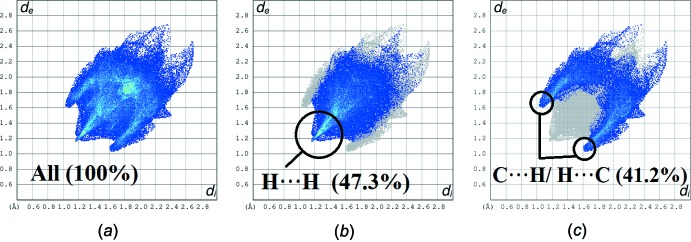
Fingerprint plots of inter­actions, listing the percentage of contacts (*a*) full two-dimensional fingerprint plots, and (*b*) H⋯H and (*c*) C⋯H/H⋯C contributions to the total Hirshfeld surface. The outline of the full fingerprint plots is shown in grey.

The two-dimensional fingerprint plots shown in Fig. 6 ▸ illustrate the difference between the inter­molecular inter­action patterns and the major inter­molecular contacts associated with the title compound. The H⋯H contacts (Fig. 6 ▸ *b*) appear to be the major contributor to the Hirshfeld surface and are seen as one distinct spike with a minimum value for *d*_e_ + *d*_i_ that is less than the sum of the van der Waals radii (2.4 Å). The inter­molecular C—H⋯π inter­actions are characterized by the short inter­atomic C⋯H/H⋯C (41.2%) contacts and their presence is indicated by the distribution of points around a pair of wings at *d*_e_ + *d*_i_ ∼2.6 Å (Fig. 6 ▸ *c*).

## Database survey   

A survey of the Cambridge Structural Database (CSD, Version 5.38, last update Nov 2016; Groom *et al.*, 2016 ▸) revealed fused-ring substituted chalcones similar to the title compound. There are four compounds which have an anthracene-ketone subtituent on the chalcone: 9-anthryl styryl ketone and 9,10-anthryl bis­(styryl ketone) (Harlow *et al.*, 1975 ▸), (2*E*)-1-(anthracen-9-yl)-3-[4-(propan-2-yl)phen­yl]prop-2-en-1-one (Girisha *et al.*, 2016 ▸) and (*E*)-1-(anthracen-9-yl)-3-(2-chloro-6-fluoro­phen­yl) prop-2-en-1-one (Abdullah *et al.*, 2016 ▸). Jung *et al.* (2008 ▸) reported two ferrocenyl chalcones containing an anthracenyl subtituent, 9-(2-ferrocenyl­ethen­yl­carbon­yl)anthracene and 1-(9-anthracen­yl)-3-ferrocenyl-2-propen-1-one. Other related compounds include, 1-(anth­rac­en-9-yl)-2-meth­ylprop-2-en-1-one (Agrahari *et al.*, 2015 ▸) and 9-anthroylacetone (Cicogna *et al.*, 2004 ▸).

## Synthesis and crystallization   

A mixture of 9-acetyl­anthracene (0.5 mmol) and 9-anthracenecarboxaldehyde (0.5 mmol) was dissolved in methanol (20 ml). A catalytic amount of NaOH (5 ml, 20%) was added to the solution dropwise with vigorous stirring. The reaction mixture was stirred for about 5-6 h at room temperature. After stirring, the contents of the flask were poured into ice-cold water (50 ml). The resultant crude products were filtered, washed successively with distilled water and recrystallized from acetone solution as yellow blocks. The single crystal (Fig. S4) used for data collection was obtained by the slow-evaporation technique using acetone as the solvent.

## Refinement   

Crystal data collection and structure refinement details are summarized in Table 2 ▸. All H atoms were positioned geometrically (C—H =0.93 Å) and refined using riding model with *U*_iso_(H)=1.2*U*_eq_(C).

## Supplementary Material

Crystal structure: contains datablock(s) I. DOI: 10.1107/S2056989018003791/hb7739sup1.cif

Structure factors: contains datablock(s) I. DOI: 10.1107/S2056989018003791/hb7739Isup2.hkl

Supplemetary figures and table. DOI: 10.1107/S2056989018003791/hb7739sup3.pdf

Supporting information file. DOI: 10.1107/S2056989018003791/hb7739Isup4.cml

CCDC reference: 1817217

Additional supporting information:  crystallographic information; 3D view; checkCIF report

## Figures and Tables

**Table 1:** Hydrogen-bond geometry (Å, °) *Cg*4 and *Cg*6 are the centroids of the C18/C19/C24–C26/C31 and C26–C31 rings, respectively.

*D*—H⋯*A*	*D*—H	H⋯*A*	*D*⋯*A*	*D*—H⋯*A*
C5—H5*A*⋯*Cg*4^i^	0.93	2.75	3.511 (2)	140
C7—H7*A*⋯*Cg*6^i^	0.93	2.91	3.672 (2)	140

Symmetry code: (i) 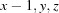.

**Table 2:** Experimental details

Crystal data	
Chemical formula	C_31_H_20_O
*M*_r_	408.47
Crystal system, space group	Triclinic, *P* 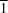
Temperature (K)	296
*a*, *b*, *c* (Å)	9.8310 (17), 10.7521 (18), 11.3029 (19)
α, β, γ (°)	67.146 (2), 73.586 (2), 78.768 (2)
*V* (Å^3^)	1051.2 (3)
*Z*	2
Radiation type	Mo *K*α
μ (mm^−1^)	0.08
Crystal size (mm)	0.45 × 0.38 × 0.26

Data collection	
Diffractometer	Bruker SMART APEXII DUO CCD area-detector
Absorption correction	Multi-scan (*SADABS*; Bruker, 2009 ▸)
No. of measured, independent and observed [*I* > 2σ(*I*)] reflections	42832, 6216, 2792
*R*_int_	0.047
(sin θ/λ)_max_ (Å^−1^)	0.709

Refinement	
*R*[*F* ^2^ > 2σ(*F* ^2^)], *wR*(*F* ^2^), *S*	0.057, 0.187, 1.00
No. of reflections	6216
No. of parameters	289
H-atom treatment	H-atom parameters constrained
Δρ_max_, Δρ_min_ (e Å^−3^)	0.20, −0.15

Computer programs: *APEX2* and *SAINT* (Bruker, 2009 ▸), *SHELXL2013* (Sheldrick, 2015 ▸), *SHELXTL* (Sheldrick, 2008 ▸) and *PLATON* (Spek, 2009 ▸).

## supplementary crystallographic information

### Crystal data

**Table:**

C_31_H_20_O	*Z* = 2
*M**_r_* = 408.47	*F*(000) = 428
Triclinic, *P*1	*D*_x_ = 1.290 Mg m^−^^3^
*a* = 9.8310 (17) Å	Mo *K*α radiation, λ = 0.71073 Å
*b* = 10.7521 (18) Å	Cell parameters from 3766 reflections
*c* = 11.3029 (19) Å	θ = 2.3–22.1°
α = 67.146 (2)°	µ = 0.08 mm^−^^1^
β = 73.586 (2)°	*T* = 296 K
γ = 78.768 (2)°	Block, yellow
*V* = 1051.2 (3) Å^3^	0.45 × 0.38 × 0.26 mm

### Data collection

**Table:**

Bruker SMART APEXII DUO CCD area-detector diffractometer	2792 reflections with *I* > 2σ(*I*)
Radiation source: fine-focus sealed tube	*R*_int_ = 0.047
φ and ω scans	θ_max_ = 30.3°, θ_min_ = 2.0°
Absorption correction: multi-scan (*SADABS*; Bruker, 2009)	*h* = −13→13
	*k* = −15→15
42832 measured reflections	*l* = −15→15
6216 independent reflections	

### Refinement

**Table:**

Refinement on *F*^2^	0 restraints
Least-squares matrix: full	Hydrogen site location: inferred from neighbouring sites
*R*[*F*^2^ > 2σ(*F*^2^)] = 0.057	H-atom parameters constrained
*wR*(*F*^2^) = 0.187	*w* = 1/[σ^2^(*F*_o_^2^) + (0.0746*P*)^2^ + 0.1037*P*] where *P* = (*F*_o_^2^ + 2*F*_c_^2^)/3
*S* = 1.00	(Δ/σ)_max_ < 0.001
6216 reflections	Δρ_max_ = 0.20 e Å^−^^3^
289 parameters	Δρ_min_ = −0.15 e Å^−^^3^

### Special details

**Table:**

Geometry. All esds (except the esd in the dihedral angle between two l.s. planes) are estimated using the full covariance matrix. The cell esds are taken into account individually in the estimation of esds in distances, angles and torsion angles; correlations between esds in cell parameters are only used when they are defined by crystal symmetry. An approximate (isotropic) treatment of cell esds is used for estimating esds involving l.s. planes.

### Fractional atomic coordinates and isotropic or equivalent isotropic displacement parameters (Å^2^)

**Table:**

	*x*	*y*	*z*	*U*_iso_*/*U*_eq_	
O1	0.44003 (14)	0.39564 (13)	0.83904 (17)	0.0974 (5)	
C1	0.18125 (17)	0.58896 (16)	0.72383 (17)	0.0557 (4)	
C2	0.2456 (2)	0.55577 (19)	0.6096 (2)	0.0745 (5)	
H2A	0.3398	0.5184	0.5977	0.089*	
C3	0.1725 (3)	0.5774 (2)	0.5176 (2)	0.0895 (6)	
H3A	0.2174	0.5560	0.4428	0.107*	
C4	0.0287 (3)	0.6321 (2)	0.5335 (2)	0.0848 (6)	
H4A	−0.0202	0.6472	0.4690	0.102*	
C5	−0.0372 (2)	0.66204 (17)	0.6411 (2)	0.0702 (5)	
H5A	−0.1325	0.6962	0.6513	0.084*	
C6	0.03472 (17)	0.64307 (16)	0.74020 (18)	0.0564 (4)	
C7	−0.03088 (16)	0.67773 (16)	0.84986 (18)	0.0589 (4)	
H7A	−0.1270	0.7093	0.8621	0.071*	
C8	0.04251 (16)	0.66686 (15)	0.94246 (17)	0.0551 (4)	
C9	−0.0227 (2)	0.70868 (18)	1.05166 (19)	0.0707 (5)	
H9A	−0.1189	0.7397	1.0651	0.085*	
C10	0.0525 (3)	0.7042 (2)	1.1362 (2)	0.0834 (6)	
H10A	0.0083	0.7335	1.2065	0.100*	
C11	0.1973 (2)	0.6556 (2)	1.1191 (2)	0.0776 (5)	
H11A	0.2487	0.6544	1.1772	0.093*	
C12	0.26246 (19)	0.61070 (18)	1.01937 (18)	0.0659 (5)	
H12A	0.3577	0.5762	1.0115	0.079*	
C13	0.18947 (16)	0.61474 (15)	0.92571 (16)	0.0529 (4)	
C14	0.25475 (16)	0.57424 (15)	0.81840 (16)	0.0528 (4)	
C15	0.40796 (17)	0.51694 (18)	0.80046 (18)	0.0633 (5)	
C16	0.51726 (17)	0.61184 (17)	0.73382 (18)	0.0666 (5)	
H16A	0.6119	0.5756	0.7213	0.080*	
C17	0.49221 (16)	0.74227 (16)	0.69108 (16)	0.0564 (4)	
H17A	0.3972	0.7775	0.7032	0.068*	
C18	0.59990 (15)	0.84051 (15)	0.62495 (16)	0.0510 (4)	
C19	0.65765 (16)	0.87661 (16)	0.48951 (17)	0.0547 (4)	
C20	0.6174 (2)	0.82323 (19)	0.40902 (19)	0.0701 (5)	
H20A	0.5499	0.7606	0.4473	0.084*	
C21	0.6744 (2)	0.8611 (2)	0.2788 (2)	0.0881 (6)	
H21A	0.6459	0.8247	0.2282	0.106*	
C22	0.7768 (3)	0.9552 (2)	0.2183 (2)	0.0955 (7)	
H22A	0.8155	0.9807	0.1280	0.115*	
C23	0.8190 (2)	1.0082 (2)	0.2896 (2)	0.0830 (6)	
H23A	0.8875	1.0697	0.2480	0.100*	
C24	0.76171 (18)	0.97267 (17)	0.42761 (18)	0.0634 (5)	
C25	0.80165 (19)	1.02850 (18)	0.5019 (2)	0.0720 (5)	
H25A	0.8687	1.0914	0.4605	0.086*	
C26	0.74538 (19)	0.99421 (17)	0.6362 (2)	0.0658 (5)	
C27	0.7864 (3)	1.0506 (2)	0.7140 (3)	0.0899 (7)	
H27A	0.8524	1.1146	0.6740	0.108*	
C28	0.7316 (3)	1.0133 (2)	0.8443 (3)	0.0980 (7)	
H28A	0.7605	1.0513	0.8933	0.118*	
C29	0.6313 (2)	0.9178 (2)	0.9075 (2)	0.0841 (6)	
H29A	0.5946	0.8925	0.9981	0.101*	
C30	0.58805 (19)	0.86234 (18)	0.83782 (19)	0.0676 (5)	
H30A	0.5211	0.7994	0.8812	0.081*	
C31	0.64201 (16)	0.89751 (15)	0.70003 (17)	0.0555 (4)	

### Atomic displacement parameters (Å^2^)

**Table:**

	*U*^11^	*U*^22^	*U*^33^	*U*^12^	*U*^13^	*U*^23^
O1	0.0731 (9)	0.0536 (8)	0.1465 (14)	−0.0020 (6)	−0.0287 (9)	−0.0149 (8)
C1	0.0544 (9)	0.0476 (9)	0.0641 (11)	−0.0107 (7)	−0.0152 (8)	−0.0149 (8)
C2	0.0757 (12)	0.0749 (13)	0.0775 (13)	−0.0077 (10)	−0.0177 (10)	−0.0317 (11)
C3	0.1108 (18)	0.0908 (16)	0.0807 (15)	−0.0134 (13)	−0.0278 (13)	−0.0390 (12)
C4	0.1070 (17)	0.0767 (14)	0.0872 (16)	−0.0140 (12)	−0.0502 (14)	−0.0245 (12)
C5	0.0702 (11)	0.0582 (11)	0.0882 (14)	−0.0115 (8)	−0.0370 (11)	−0.0167 (10)
C6	0.0543 (9)	0.0445 (9)	0.0702 (11)	−0.0120 (7)	−0.0225 (8)	−0.0109 (8)
C7	0.0435 (8)	0.0515 (9)	0.0758 (12)	−0.0082 (7)	−0.0146 (8)	−0.0140 (8)
C8	0.0526 (9)	0.0454 (9)	0.0602 (10)	−0.0105 (7)	−0.0106 (8)	−0.0099 (7)
C9	0.0670 (11)	0.0653 (12)	0.0684 (12)	−0.0033 (9)	−0.0078 (10)	−0.0186 (9)
C10	0.1013 (16)	0.0763 (14)	0.0656 (13)	−0.0026 (12)	−0.0139 (12)	−0.0242 (10)
C11	0.0948 (15)	0.0772 (13)	0.0635 (12)	−0.0098 (11)	−0.0296 (11)	−0.0190 (10)
C12	0.0628 (10)	0.0655 (11)	0.0650 (12)	−0.0092 (8)	−0.0222 (9)	−0.0118 (9)
C13	0.0485 (8)	0.0464 (9)	0.0583 (10)	−0.0116 (7)	−0.0135 (7)	−0.0086 (7)
C14	0.0454 (8)	0.0484 (9)	0.0593 (10)	−0.0095 (6)	−0.0121 (7)	−0.0111 (8)
C15	0.0544 (9)	0.0546 (10)	0.0756 (12)	−0.0044 (8)	−0.0167 (8)	−0.0166 (9)
C16	0.0412 (8)	0.0584 (11)	0.0867 (13)	0.0004 (7)	−0.0092 (8)	−0.0175 (9)
C17	0.0419 (8)	0.0567 (10)	0.0652 (11)	−0.0010 (7)	−0.0133 (7)	−0.0171 (8)
C18	0.0399 (7)	0.0478 (9)	0.0582 (10)	0.0023 (6)	−0.0125 (7)	−0.0134 (7)
C19	0.0481 (8)	0.0498 (9)	0.0575 (10)	0.0041 (7)	−0.0113 (7)	−0.0144 (8)
C20	0.0672 (11)	0.0724 (12)	0.0659 (12)	0.0017 (9)	−0.0162 (9)	−0.0228 (10)
C21	0.0967 (16)	0.0941 (16)	0.0701 (14)	0.0103 (13)	−0.0219 (12)	−0.0325 (12)
C22	0.1056 (18)	0.0903 (16)	0.0582 (13)	0.0117 (13)	−0.0007 (12)	−0.0146 (12)
C23	0.0759 (13)	0.0669 (13)	0.0733 (14)	−0.0007 (10)	0.0034 (11)	−0.0072 (11)
C24	0.0568 (10)	0.0500 (10)	0.0634 (11)	0.0030 (8)	−0.0074 (8)	−0.0071 (8)
C25	0.0629 (11)	0.0528 (10)	0.0829 (14)	−0.0129 (8)	−0.0105 (10)	−0.0064 (10)
C26	0.0638 (10)	0.0507 (10)	0.0796 (13)	−0.0066 (8)	−0.0209 (10)	−0.0160 (9)
C27	0.1027 (16)	0.0627 (13)	0.1131 (19)	−0.0197 (11)	−0.0405 (15)	−0.0240 (13)
C28	0.126 (2)	0.0814 (15)	0.109 (2)	−0.0107 (14)	−0.0510 (17)	−0.0402 (14)
C29	0.0993 (16)	0.0847 (15)	0.0747 (14)	0.0017 (12)	−0.0278 (12)	−0.0343 (12)
C30	0.0658 (11)	0.0691 (12)	0.0658 (12)	−0.0019 (9)	−0.0154 (9)	−0.0235 (9)
C31	0.0509 (9)	0.0493 (9)	0.0624 (11)	0.0010 (7)	−0.0161 (8)	−0.0162 (8)

### Geometric parameters (Å, º)

**Table:**

O1—C15	1.2109 (19)	C16—H16A	0.9300
C1—C14	1.397 (2)	C17—C18	1.473 (2)
C1—C2	1.416 (3)	C17—H17A	0.9300
C1—C6	1.433 (2)	C18—C19	1.396 (2)
C2—C3	1.349 (3)	C18—C31	1.403 (2)
C2—H2A	0.9300	C19—C20	1.418 (3)
C3—C4	1.411 (3)	C19—C24	1.431 (2)
C3—H3A	0.9300	C20—C21	1.343 (3)
C4—C5	1.333 (3)	C20—H20A	0.9300
C4—H4A	0.9300	C21—C22	1.406 (3)
C5—C6	1.418 (2)	C21—H21A	0.9300
C5—H5A	0.9300	C22—C23	1.335 (3)
C6—C7	1.380 (2)	C22—H22A	0.9300
C7—C8	1.389 (2)	C23—C24	1.422 (3)
C7—H7A	0.9300	C23—H23A	0.9300
C8—C9	1.417 (2)	C24—C25	1.374 (3)
C8—C13	1.432 (2)	C25—C26	1.385 (3)
C9—C10	1.346 (3)	C25—H25A	0.9300
C9—H9A	0.9300	C26—C27	1.419 (3)
C10—C11	1.404 (3)	C26—C31	1.431 (2)
C10—H10A	0.9300	C27—C28	1.340 (3)
C11—C12	1.344 (3)	C27—H27A	0.9300
C11—H11A	0.9300	C28—C29	1.401 (3)
C12—C13	1.421 (2)	C28—H28A	0.9300
C12—H12A	0.9300	C29—C30	1.345 (3)
C13—C14	1.391 (2)	C29—H29A	0.9300
C14—C15	1.501 (2)	C30—C31	1.416 (2)
C15—C16	1.461 (2)	C30—H30A	0.9300
C16—C17	1.291 (2)		
			
C14—C1—C2	123.06 (16)	C15—C16—H16A	117.6
C14—C1—C6	119.19 (16)	C16—C17—C18	126.16 (14)
C2—C1—C6	117.74 (16)	C16—C17—H17A	116.9
C3—C2—C1	121.13 (19)	C18—C17—H17A	116.9
C3—C2—H2A	119.4	C19—C18—C31	120.92 (15)
C1—C2—H2A	119.4	C19—C18—C17	120.16 (15)
C2—C3—C4	120.9 (2)	C31—C18—C17	118.92 (15)
C2—C3—H3A	119.6	C18—C19—C20	123.09 (16)
C4—C3—H3A	119.6	C18—C19—C24	119.04 (16)
C5—C4—C3	120.04 (19)	C20—C19—C24	117.87 (16)
C5—C4—H4A	120.0	C21—C20—C19	121.5 (2)
C3—C4—H4A	120.0	C21—C20—H20A	119.3
C4—C5—C6	121.60 (19)	C19—C20—H20A	119.3
C4—C5—H5A	119.2	C20—C21—C22	120.5 (2)
C6—C5—H5A	119.2	C20—C21—H21A	119.7
C7—C6—C5	122.16 (16)	C22—C21—H21A	119.7
C7—C6—C1	119.23 (15)	C23—C22—C21	120.5 (2)
C5—C6—C1	118.59 (18)	C23—C22—H22A	119.8
C6—C7—C8	121.90 (15)	C21—C22—H22A	119.8
C6—C7—H7A	119.0	C22—C23—C24	121.5 (2)
C8—C7—H7A	119.0	C22—C23—H23A	119.2
C7—C8—C9	122.02 (16)	C24—C23—H23A	119.2
C7—C8—C13	119.12 (16)	C25—C24—C23	122.26 (19)
C9—C8—C13	118.85 (16)	C25—C24—C19	119.60 (17)
C10—C9—C8	121.02 (18)	C23—C24—C19	118.14 (19)
C10—C9—H9A	119.5	C24—C25—C26	122.21 (17)
C8—C9—H9A	119.5	C24—C25—H25A	118.9
C9—C10—C11	120.5 (2)	C26—C25—H25A	118.9
C9—C10—H10A	119.8	C25—C26—C27	122.71 (19)
C11—C10—H10A	119.8	C25—C26—C31	118.98 (17)
C12—C11—C10	120.56 (19)	C27—C26—C31	118.31 (19)
C12—C11—H11A	119.7	C28—C27—C26	121.1 (2)
C10—C11—H11A	119.7	C28—C27—H27A	119.4
C11—C12—C13	121.64 (18)	C26—C27—H27A	119.4
C11—C12—H12A	119.2	C27—C28—C29	120.8 (2)
C13—C12—H12A	119.2	C27—C28—H28A	119.6
C14—C13—C12	123.25 (15)	C29—C28—H28A	119.6
C14—C13—C8	119.31 (15)	C30—C29—C28	120.3 (2)
C12—C13—C8	117.42 (16)	C30—C29—H29A	119.9
C13—C14—C1	121.16 (15)	C28—C29—H29A	119.9
C13—C14—C15	120.13 (15)	C29—C30—C31	121.57 (19)
C1—C14—C15	118.69 (15)	C29—C30—H30A	119.2
O1—C15—C16	120.98 (16)	C31—C30—H30A	119.2
O1—C15—C14	120.98 (15)	C18—C31—C30	122.86 (15)
C16—C15—C14	118.03 (14)	C18—C31—C26	119.26 (16)
C17—C16—C15	124.87 (15)	C30—C31—C26	117.88 (16)
C17—C16—H16A	117.6		
			
C14—C1—C2—C3	−176.92 (17)	C14—C15—C16—C17	1.4 (3)
C6—C1—C2—C3	1.6 (3)	C15—C16—C17—C18	179.28 (17)
C1—C2—C3—C4	−0.9 (3)	C16—C17—C18—C19	84.0 (2)
C2—C3—C4—C5	−0.6 (3)	C16—C17—C18—C31	−96.6 (2)
C3—C4—C5—C6	1.4 (3)	C31—C18—C19—C20	−179.19 (14)
C4—C5—C6—C7	177.88 (16)	C17—C18—C19—C20	0.3 (2)
C4—C5—C6—C1	−0.7 (3)	C31—C18—C19—C24	0.3 (2)
C14—C1—C6—C7	−0.8 (2)	C17—C18—C19—C24	179.77 (14)
C2—C1—C6—C7	−179.38 (14)	C18—C19—C20—C21	179.48 (16)
C14—C1—C6—C5	177.76 (14)	C24—C19—C20—C21	0.0 (3)
C2—C1—C6—C5	−0.8 (2)	C19—C20—C21—C22	0.2 (3)
C5—C6—C7—C8	−175.90 (14)	C20—C21—C22—C23	0.1 (3)
C1—C6—C7—C8	2.6 (2)	C21—C22—C23—C24	−0.5 (3)
C6—C7—C8—C9	176.81 (14)	C22—C23—C24—C25	−178.53 (18)
C6—C7—C8—C13	−1.7 (2)	C22—C23—C24—C19	0.7 (3)
C7—C8—C9—C10	−176.32 (16)	C18—C19—C24—C25	−0.7 (2)
C13—C8—C9—C10	2.2 (3)	C20—C19—C24—C25	178.85 (15)
C8—C9—C10—C11	−1.1 (3)	C18—C19—C24—C23	−179.92 (15)
C9—C10—C11—C12	−1.1 (3)	C20—C19—C24—C23	−0.4 (2)
C10—C11—C12—C13	2.1 (3)	C23—C24—C25—C26	179.77 (16)
C11—C12—C13—C14	177.40 (16)	C19—C24—C25—C26	0.6 (3)
C11—C12—C13—C8	−0.9 (2)	C24—C25—C26—C27	179.65 (17)
C7—C8—C13—C14	−1.0 (2)	C24—C25—C26—C31	−0.1 (3)
C9—C8—C13—C14	−179.59 (14)	C25—C26—C27—C28	−178.81 (19)
C7—C8—C13—C12	177.34 (14)	C31—C26—C27—C28	0.9 (3)
C9—C8—C13—C12	−1.2 (2)	C26—C27—C28—C29	−0.3 (4)
C12—C13—C14—C1	−175.44 (14)	C27—C28—C29—C30	−0.3 (3)
C8—C13—C14—C1	2.8 (2)	C28—C29—C30—C31	0.4 (3)
C12—C13—C14—C15	2.9 (2)	C19—C18—C31—C30	−178.97 (14)
C8—C13—C14—C15	−178.87 (14)	C17—C18—C31—C30	1.6 (2)
C2—C1—C14—C13	176.59 (14)	C19—C18—C31—C26	0.2 (2)
C6—C1—C14—C13	−1.9 (2)	C17—C18—C31—C26	−179.29 (14)
C2—C1—C14—C15	−1.8 (2)	C29—C30—C31—C18	179.39 (16)
C6—C1—C14—C15	179.75 (14)	C29—C30—C31—C26	0.2 (3)
C13—C14—C15—O1	95.9 (2)	C25—C26—C31—C18	−0.3 (2)
C1—C14—C15—O1	−85.7 (2)	C27—C26—C31—C18	179.96 (15)
C13—C14—C15—C16	−84.7 (2)	C25—C26—C31—C30	178.88 (15)
C1—C14—C15—C16	93.65 (19)	C27—C26—C31—C30	−0.9 (2)
O1—C15—C16—C17	−179.19 (19)		

### Hydrogen-bond geometry (Å, º)

Cg4 and Cg6 are the centroids of the C18/C19/C24–C26/C31 and C26–C31 rings, respectively.

**Table:**

*D*—H···*A*	*D*—H	H···*A*	*D*···*A*	*D*—H···*A*
C5—H5*A*···*Cg*4^i^	0.93	2.75	3.511 (2)	140
C7—H7*A*···*Cg*6^i^	0.93	2.91	3.672 (2)	140

Symmetry code: (i) *x*−1, *y*, *z*.

### Comparison of experimental and calculated molecular geometry parameters (Å, °)

**Table:**

Parameters	Exp	DFT
C15—O1	1.21 (19)	1.22
C1—C14	1.40 (2)	1.40
C1—C2	1.42 (3)	1.42
C2—C3	1.35 (3)	1.35
C3—C4	1.41 (3)	1.41
C4—C5	1.33 (3)	1.33
C5—C6	1.42 (2)	1.42
C6—C7	1.380 (2)	1.38
C7—C8	1.39 (2)	1.39
C8—C9	1.42 (2)	1.42
C9—C10	1.35 (3)	1.35
C10—C11	1.40 (3)	1.40
C11—C12	1.34 (3)	1.34
C12—C13	1.42 (2)	1.42
C13—C14	1.39 (2)	1.39
C14—C15	1.50 (2)	1.52
C15—C16	1.46 (2)	1.48
C16—C17	1.29 (2)	1.35
C17—C18	1.47 (2)	1.47
C18—C19	1.40 (2)	1.40
C19—C20	1.42 (3)	1.42
C20—C21	1.34 (3)	1.34
C21—C22	1.41 (3)	1.41
C22—C23	1.34 (3)	1.34
C23—C24	1.42 (3)	1.42
C24—C25	1.37 (3)	1.37
C25—C26	1.39 (3)	1.38
C26—C27	1.42 (3)	1.42
C27—C28	1.34 (3)	1.34
C28—C29	1.40 (3)	1.40
C29—C30	1.35 (3)	1.35
C30—C31	1.42 (2)	1.42
C31—C18	1.40 (2)	1.40
C14—C15—C16	118.03 (14)	119.35
O1—C15—C14	120.98 (15)	120.25
O1—C15—C16	120.98 (16)	120.40
C15—C16—C17	124.87 (15)	123.93
C16—C17—C18	126.16 (14)	127.15
