# Supplementary material for: (E)-1,3-Bis(anthracen-9-yl)prop-2-en-1-one: crystal structure and DFT study
Source: Acta Crystallogr E Crystallogr Commun. 2018 Mar 9;74(Pt 4):492–6. doi: 10.1107/S2056989018003791 (PMC5946975; doi:10.1107/S2056989018003791)
Supplement: Supplementary file 3 [file e-74-00492-sup3.pdf]

(*E*)-1,3-di(anthrancen-9yl)prop-2-en-1-one: Crystal structure and DFT study of linear fused rings

Supplementary Materials

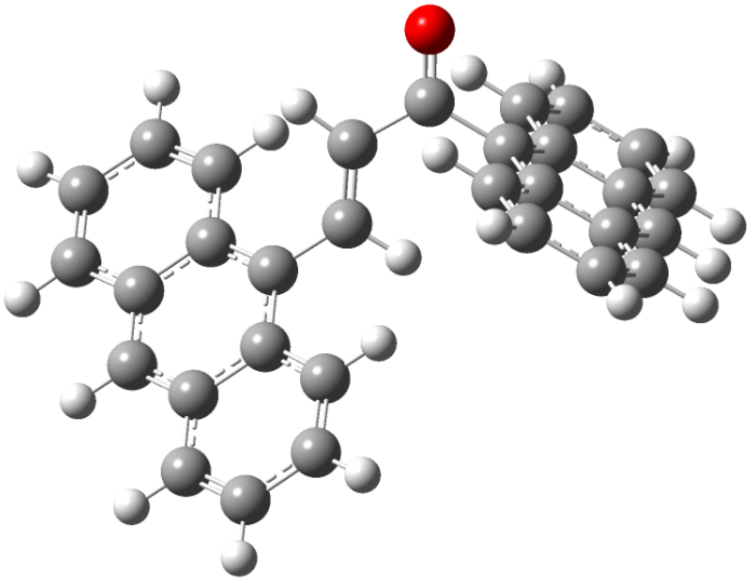

Figure S1 The optimized structure of the compound at DFT/B3LYP 6-311++G(d,p).

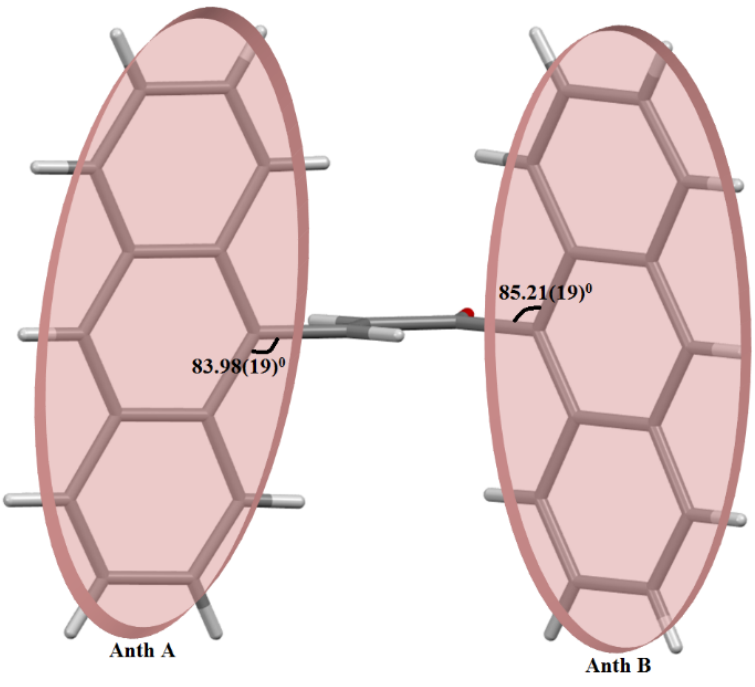

Figure S2 The twisted structure of the anthrancene units (Anth A and Anth B).

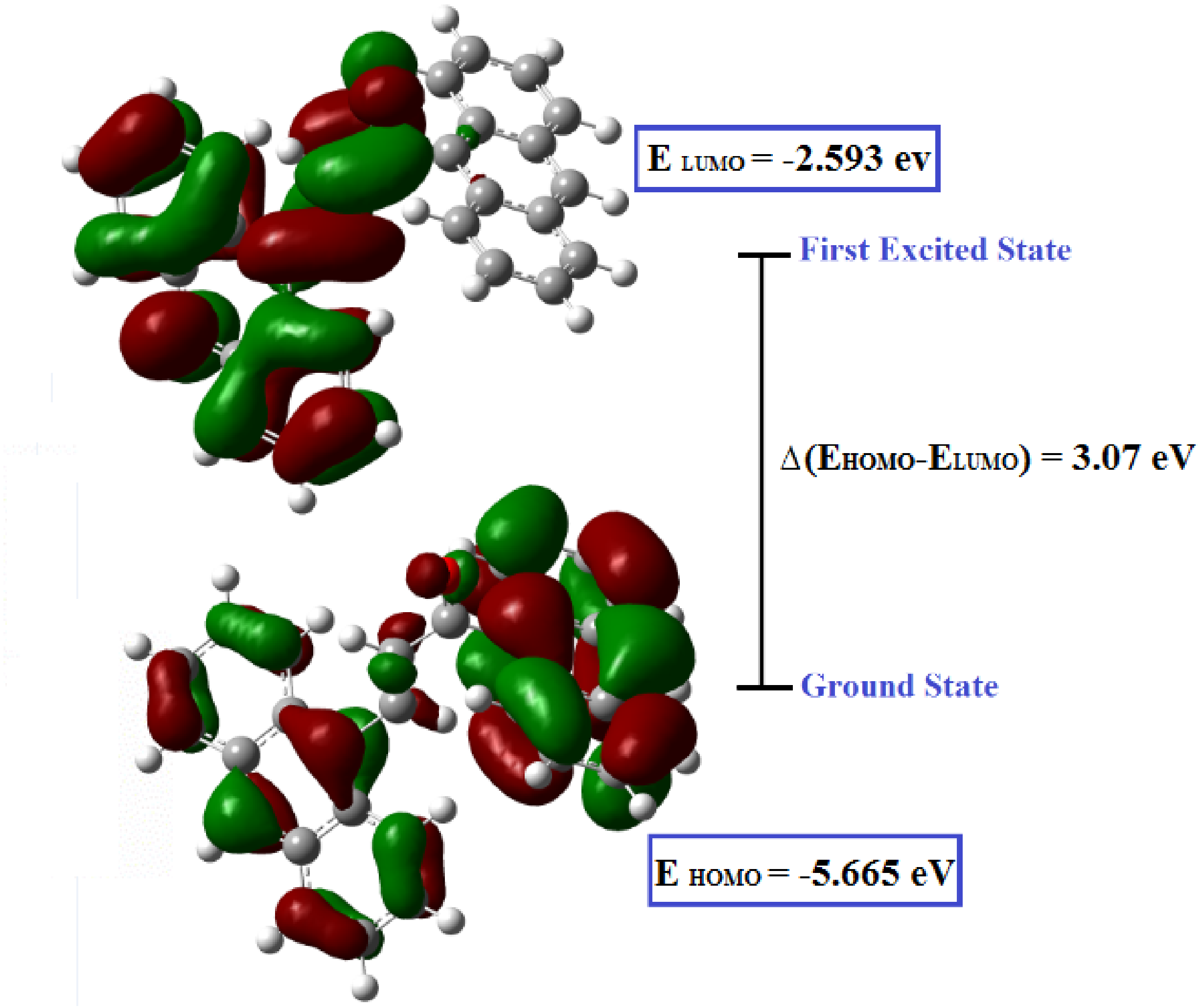

Figure S3 Electron distribution of the HOMO and LUMO energy level of the title compound.

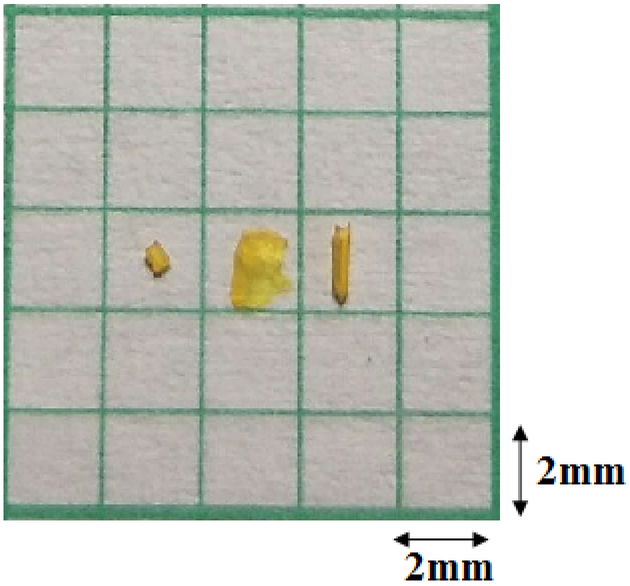

Figure S4 The single crystal of (E)-1,3-di(anthrancen-9yl)prop-2-en-1-one

Table S1 Comparison between calculated (DFT) and X-ray of selected geometrical data for title compound

Bonds	X-ray (Experimental) (Å^0)	DFT (Theoretical) (Å^0)
C15-O1	1.21 (19)	1.22
C1-C14	1.40(2)	1.40
C1-C2	1.42 (3)	1.42
C2-C3	1.35 (3)	1.35
C3-C4	1.41 (3)	1.41
C4-C5	1.33 (3)	1.33
C5-C6	1.42 (2)	1.42
C6-C7	1.380 (2)	1.38
C7-C8	1.39 (2)	1.39
C8-C9	1.42(2)	1.42
C9-C10	1.35 (3)	1.35
C10-C11	1.40 (3)	1.40
C11-C12	1.34 (3)	1.34
C12-C13	1.42 (2)	1.42
C13-C14	1.39 (2)	1.39
C14-C15	1.50 (2)	1.52
C15-C16	1.46 (2)	1.48
C16-C17	1.29 (2)	1.35
C17-C18	1.47 (2)	1.47
C18-C19	1.40 (2)	1.40

C19-C20	1.42 (3)	1.42
C20-C21	1.34 (3)	1.34
C21-C22	1.41 (3)	1.41
C22-C23	1.34 (3)	1.34
C23-C24	1.42 (3)	1.42
C24-C25	1.37 (3)	1.37
C25-C26	1.39 (3)	1.38
C26-C27	1.42 (3)	1.42
C27-C28	1.34 (3)	1.34
C28-C29	1.40 (3)	1.40
C29-C30	1.35 (3)	1.35
C30-C31	1.42 (2)	1.42
C31-C18	1.40 (2)	1.40
C14—C15—C16	118.03 (14)	119.35
O1—C15—C14	120.98 (15)	120.25
O1—C15—C16	120.98 (16)	120.40
C15—C16—C17	124.87 (15)	123.93
C16—C17—C18	126.16 (14)	127.15
